# Supplementary material for: FAP106 is an interaction hub for assembling microtubule inner proteins at the cilium inner junction
Source: Nat Commun. 2023 Aug 26;14:5225. doi: 10.1038/s41467-023-40230-z (PMC10460401; doi:10.1038/s41467-023-40230-z)
Supplement: Supplementary file 1 — Supplementary Information [file 41467_2023_40230_MOESM1_ESM.pdf]

Supplementary Figures

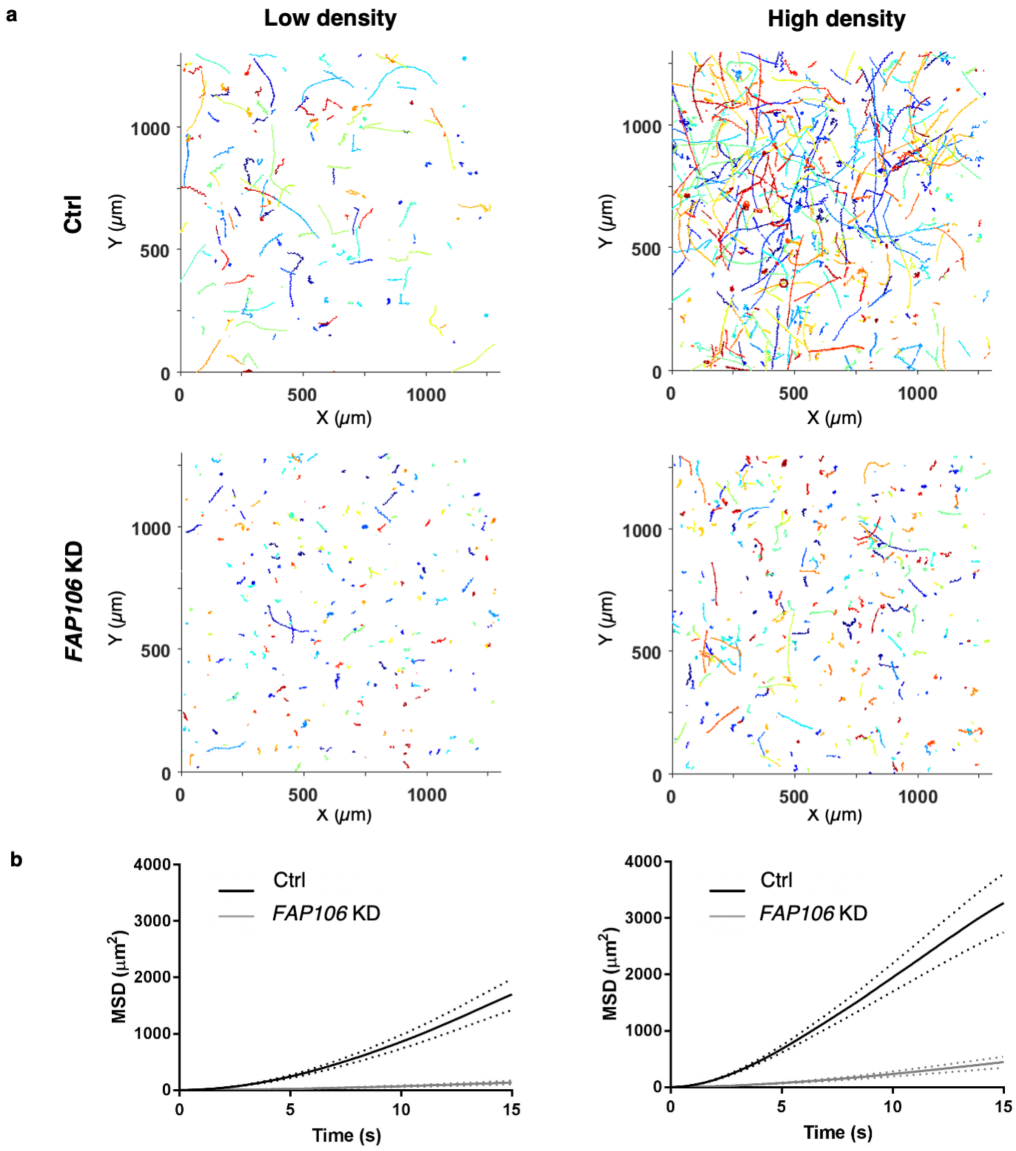

Supplementary Figure 1. Motility analysis of *FAP106* KD. a Tracks of individual 29-13 (Ctrl) or *FAP106* KD parasites grown in the presence of tetracycline to induce knockdown. Parasites were grown to a density of ~1e6 cells/ml (low density) or ~1e7 cells/ml and diluted to ~1e6 cells/ml in conditioned medium just prior to imaging (high density). **b** Mean squared displacement of parasites tracked in **a**. Dotted lines indicate the upper and lower bounds of the standard error of the mean. Low density: Ctrl N=263, *FAP106* KD N=388; High density: Ctrl N=219, *FAP106* KD N=389. Data are from an independent biological experiment from Fig. 1**f-g**.

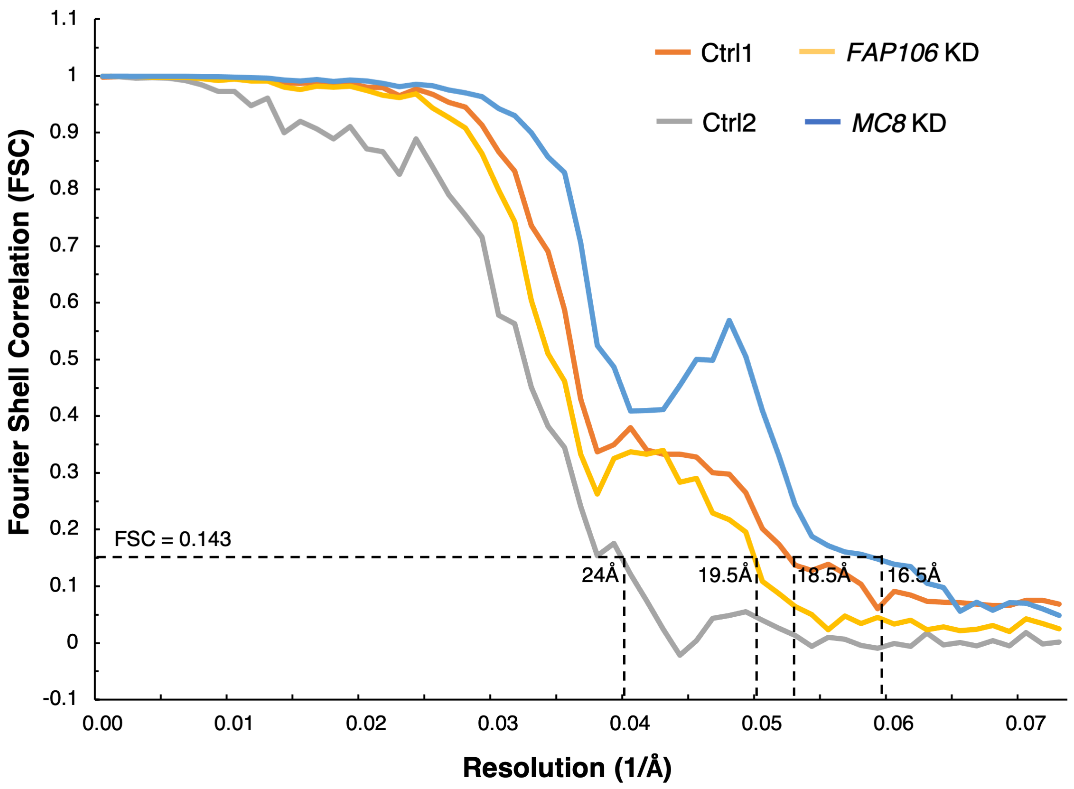

Supplementary Figure 2. Resolution evaluation of sub-tomographic averages. FSC coefficients as a function of spatial frequency for the final sub-tomographic averages of the 48-nm repeat. Ctrl1 and Ctrl2 are from 29-13, analyzed alongside *FAP106* KD (Fig. 2) and *MC8* KD (Fig. 4), respectively. The effective resolution of each structure, as indicated by the dashed lines, is based on the FSC at the 0.143 coefficient cutoff.

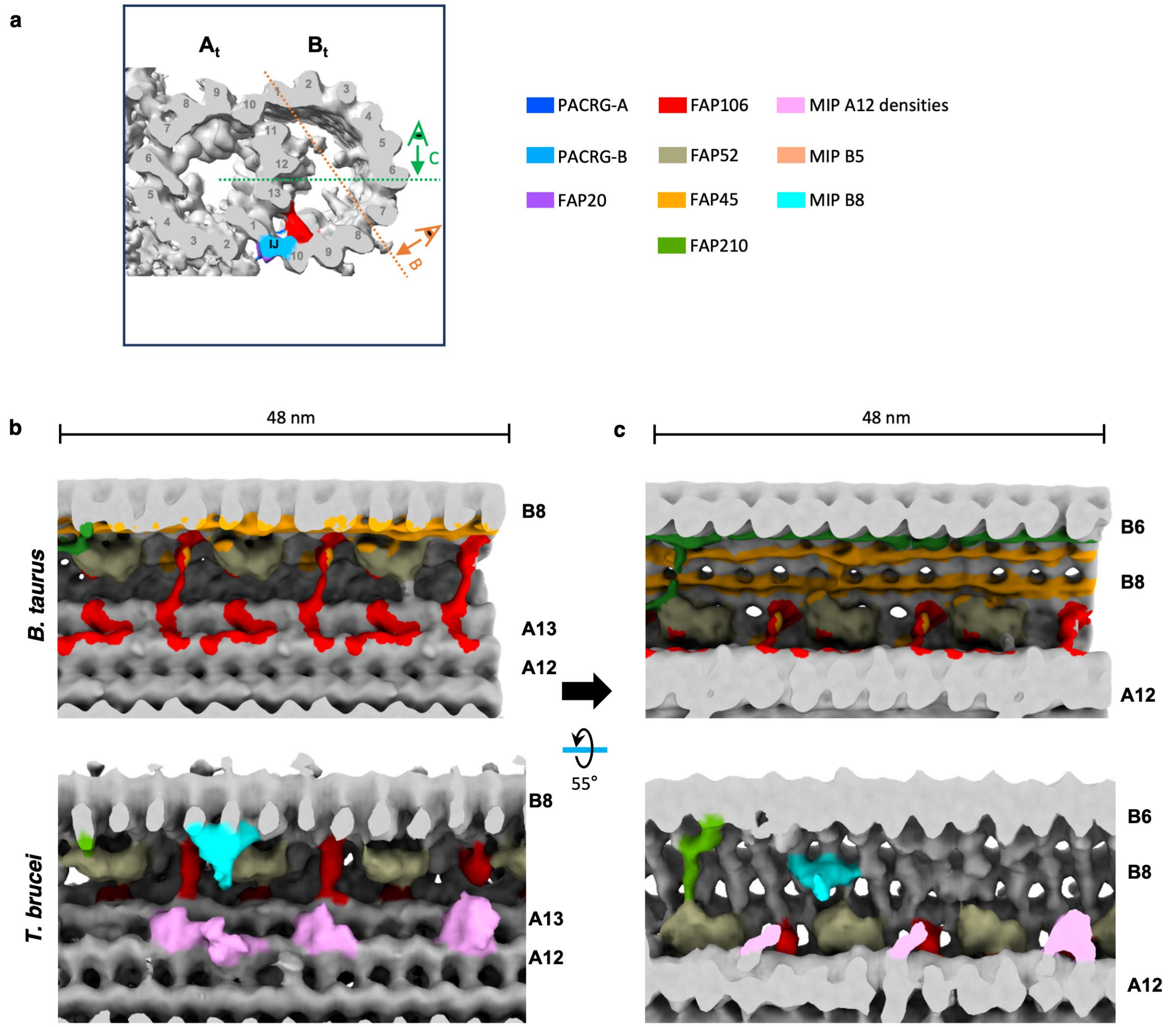

Supplementary Figure 3. Comparison of the 48-nm repeat from *T. brucei* and *B. taurus* DMTs. a Cross-sectional view of the *T. brucei* DMT indicating viewing angles shown in **b-c**. **b-c** Longitudinal views of the *T. brucei* DMT shown in Fig. 2 are aligned with corresponding views of the published density map from *B. taurus* (emdb-24664 [https://www.ebi.ac.uk/emdb/EMD-24664]; [1]), which has been low pass filtered to a similar resolution. Conserved MIP densities are colored based on similarity to densities in *B.* taurus and other published structures [1-4] as indicated by the legend. Note that *T. brucei* has a structure similar to FAP210 (green) in *B. taurus* despite not having a clear FAP210 homolog (Table 1). Lineage-specific *T. brucei* densities that are not present in the *B. taurus* structure and are reduced in *FAP106* KD (Fig. 2) are colored according to the legend.

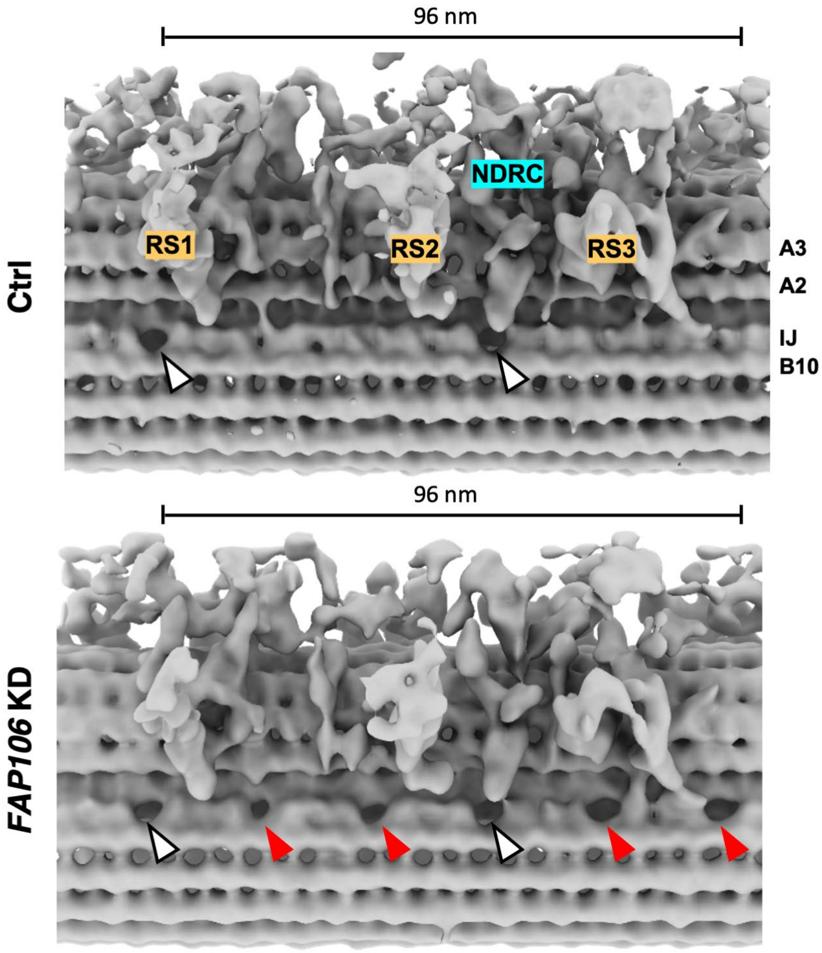

Supplementary Figure 4. Holes in the IJ filament in the 96-nm repeating unit of control and *FAP106* KD DMTs. Structure of the 96-nm repeat of the DMT was obtained by cryoET, with subtomographic averaging. Holes in the IJ filament are indicated with arrowheads. Red arrowheads indicate four additional holes in the *FAP106* KD IJ filament compared to two holes in control (white arrowheads) [5]. The nexin dynein regulatory complex (NDRC) and radial spokes (RS1-3) are labeled.

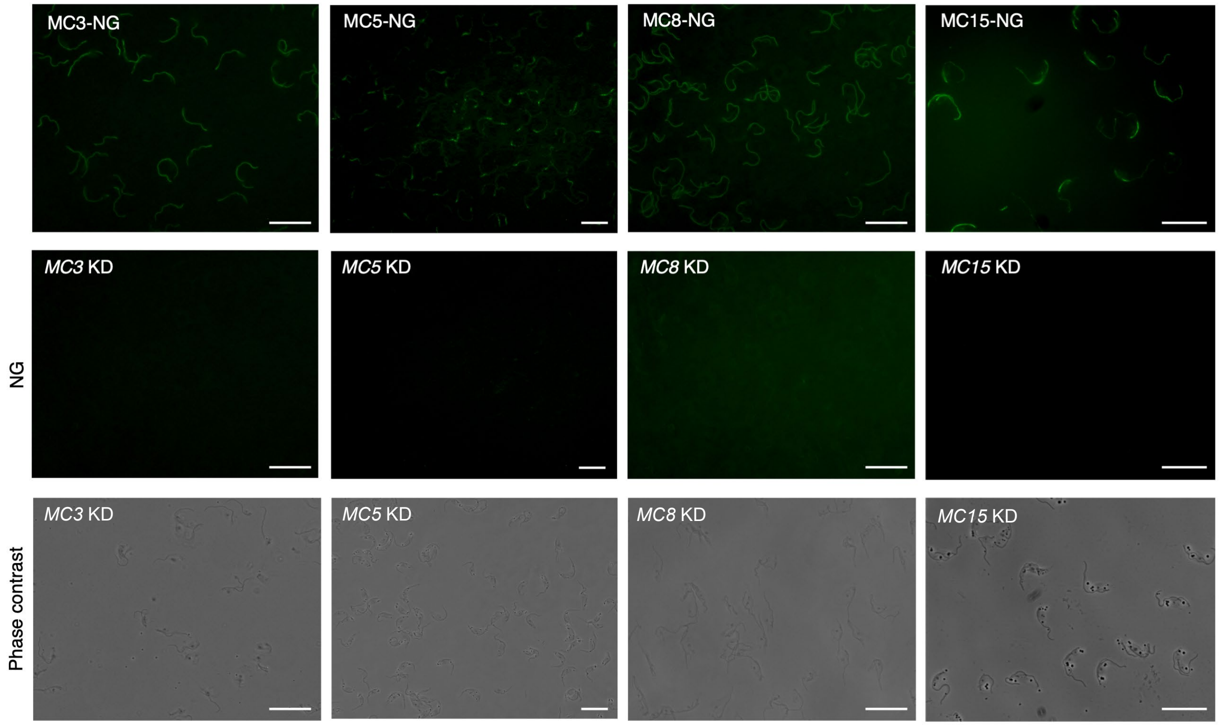

Supplementary Figure 5. Flagellar localization and knockdown of *MCs*. Fluorescence microscopy of detergent-extracted cytoskeletons shows flagellar localization of mNeonGreen (NG)-tagged MCs. Knockdown (KD) constructs were introduced into the corresponding NG-tagged lines. Parasites were grown in the presence of tetracycline to induce knockdown of the indicated *MCs*, confirming loss of flagellar localization. Each experiment was repeated independently at least twice, with similar results. Scale bar = 20 µm.

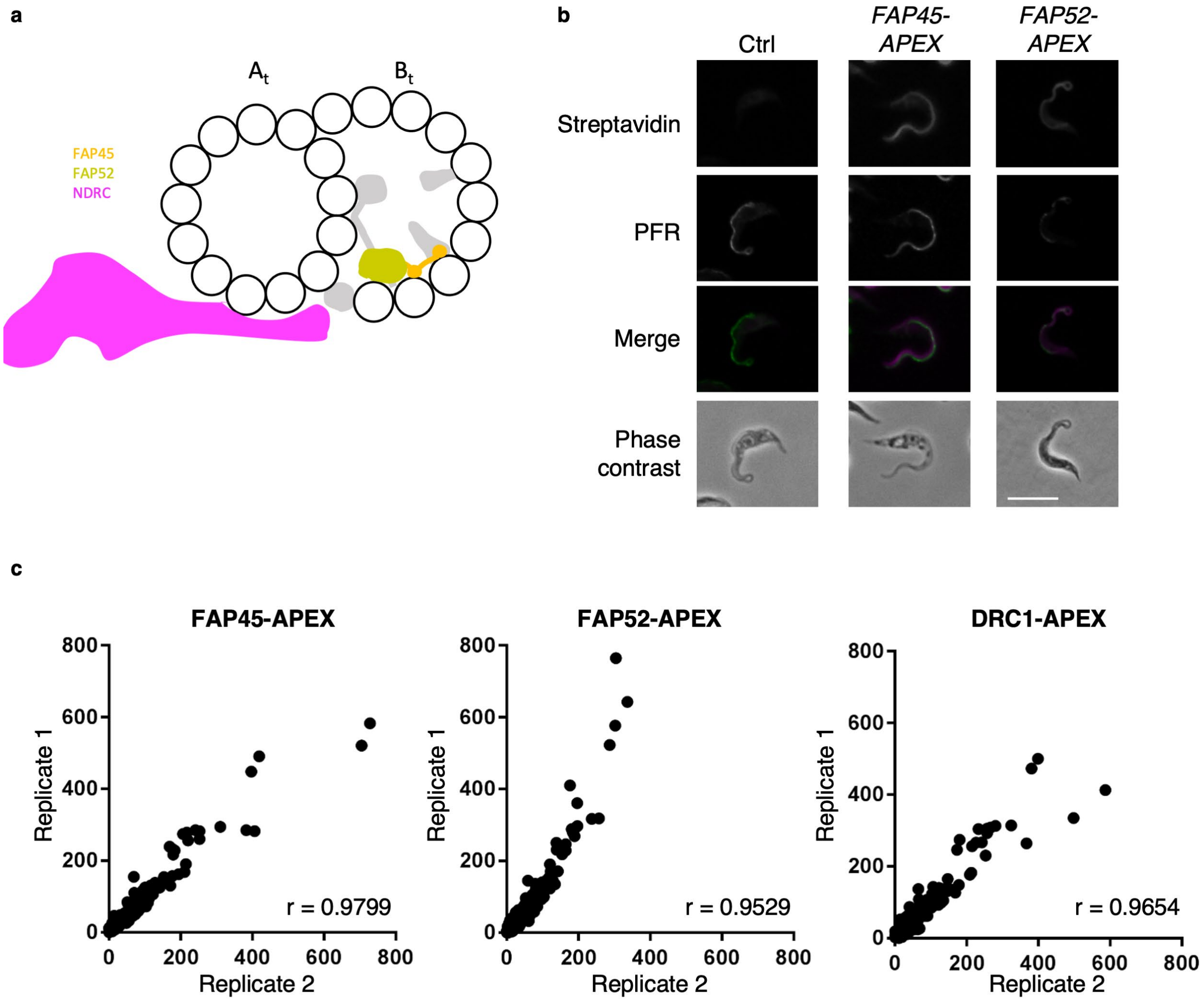

Supplementary Figure 6. APEX2-based proximity labeling of B-tubule MIPs. a Cross-sectional view of the DMT as viewed from the flagellum tip, showing generalized view of B-tubule MIPs (grey) and indicating relative positions of three different proteins tagged with APEX2 for proximity labeling: known B-tubule MIPs FAP45 (orange) and FAP52 (tan); non-MIP control outside DMT: DRC1, part of nexin-dynein regulatory comples (NDRC, pink). A- and B-tubules are labeled (A_t, B_t). **b** Immunofluorescence microscopy of whole cells showing APEX2-dependent biotinylation (streptavidin, magenta) in the flagellum (anti-PFR, green) of *FAP45-APEX* and *FAP52-APEX* cells. APEX2-dependent biotinylation in *DRC1-APEX* cells has been described previously [6]. Scale bar = 10 µm. **c** Plots showing correlation of protein abundance between independent biological replicates. Pearson coefficients (r) were calculated in GraphPad Prism.

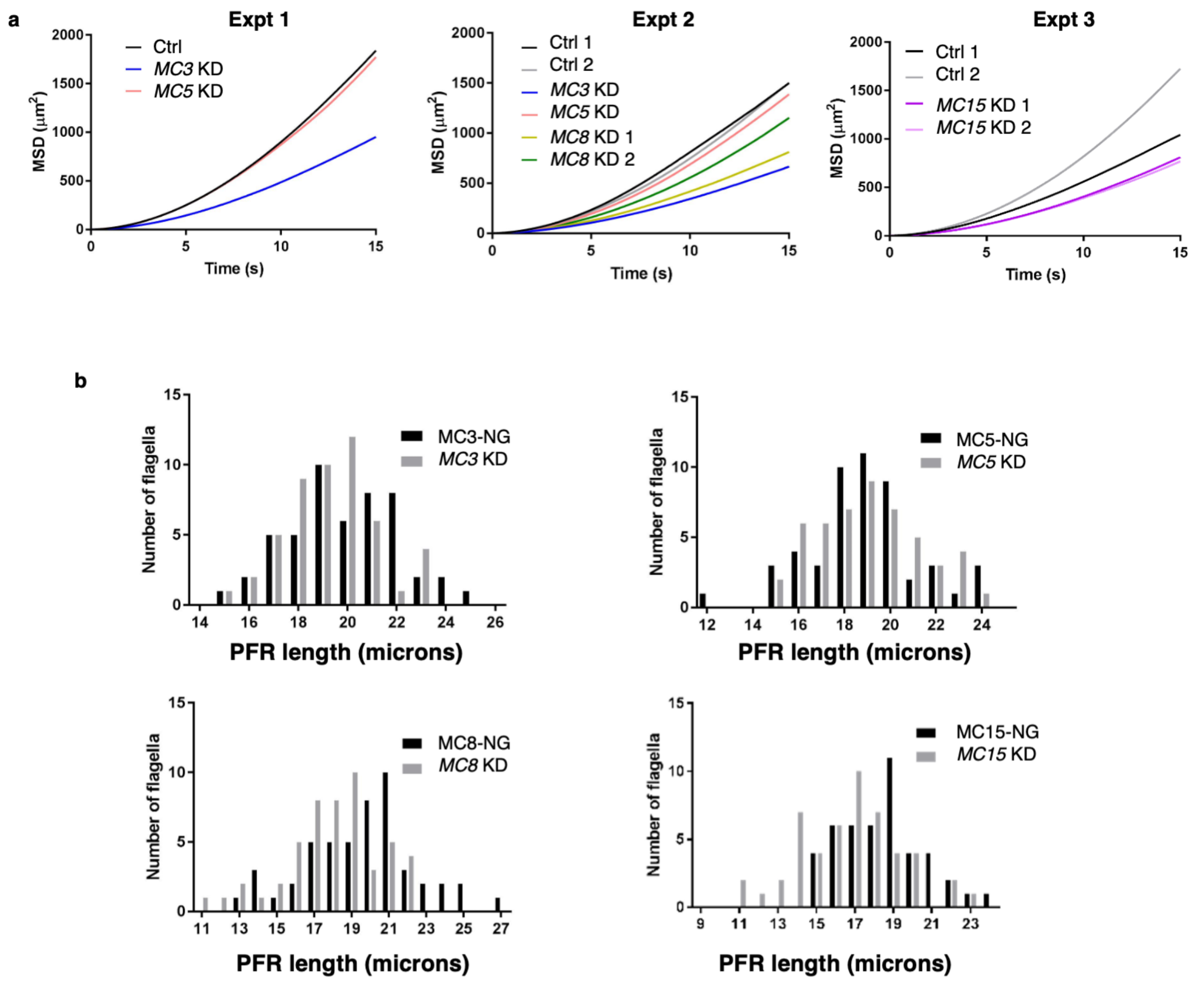

Supplementary Figure 7. Effect of *MC* knockdown on parasite motility and flagellum length. Parasites were grown in the presence of tetracycline to induce knockdown of the indicated *MC*s. (*A*) Motility analyses showing the mean squared displacement (MSD) of parasites. Expt 1: 29-13 (Ctrl) N=361, *MC3* KD N=318, *MC5* KD N=415; Expt 2: 29-13 (Ctrl 1) N=309, 29-13 (Ctrl 2) N=360, *MC3* KD N=309, *MC5* KD N=338, *MC8* KD 1 N=361, *MC8* KD 2 N=348; Expt 3: 29-13 (Ctrl 1) N=342, 29-13 (Ctrl 2) N=389, *MC15* KD 1 N=367, *MC15* KD 2 N=384. Fig. 3C shows the mean squared displacement of the three combined experiments that are shown here. **b** Detergent-extracted cytoskeletons were prepared from the indicated KD parasites or their respective NG-tagged parental cell lines (Ctrl). Histograms show the distribution of flagellum lengths as measured by anti-paraflagellar rod (PFR) immunofluorescence microscopy. Average flagellum lengths are plotted in Fig. 3**d**.

a

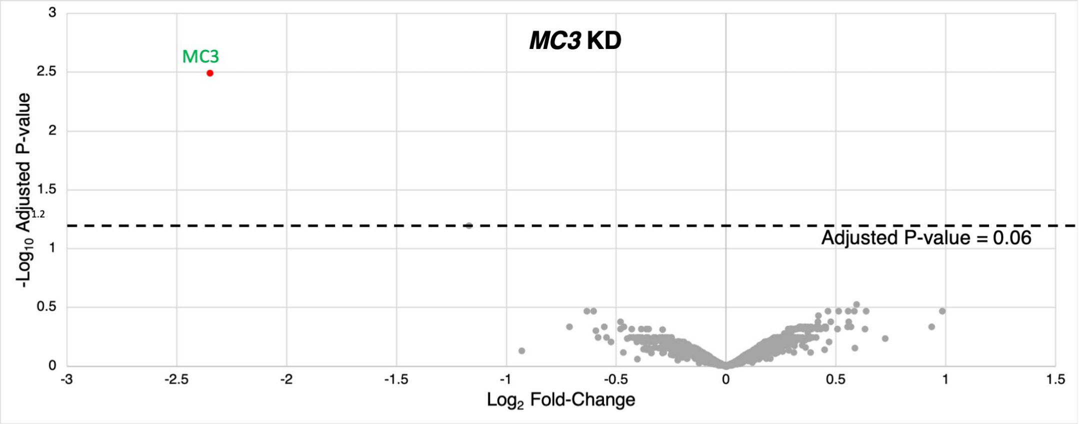

MC3 KD
MC3
Adjusted P-value = 0.06
-Log10 Adjusted P-value
Log2 Fold-Change

b

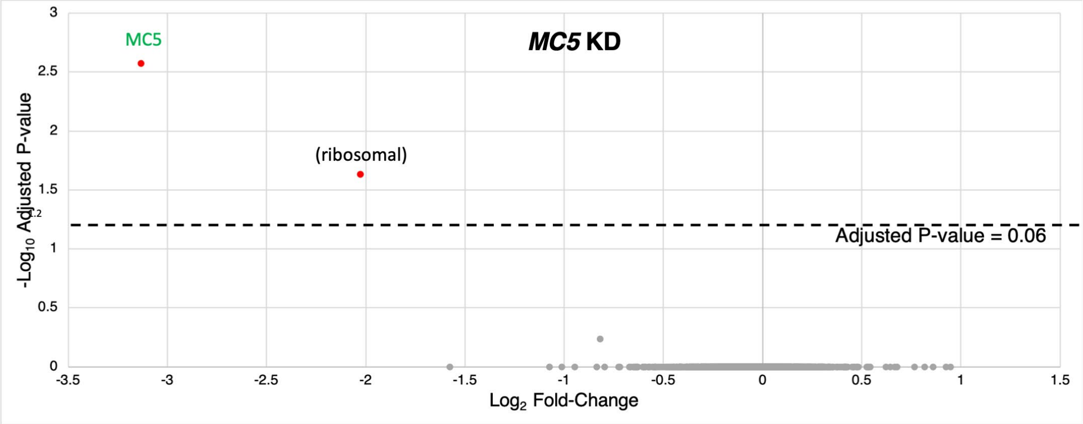

MC5 KD
MC5
(ribosomal)
Adjusted P-value = 0.06
-Log10 Adjusted P-value
Log2 Fold-Change

c

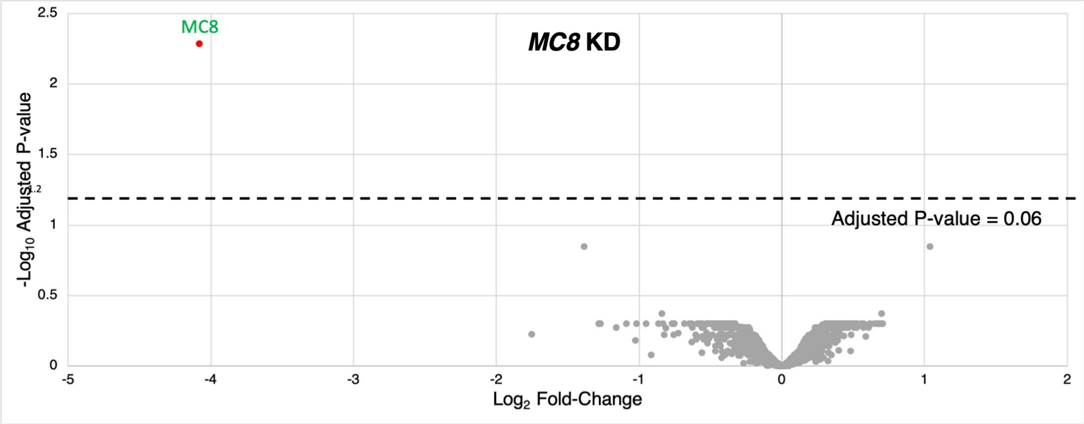

MC8 KD
MC8
Adjusted P-value = 0.06
-Log10 Adjusted P-value
Log2 Fold-Change

d

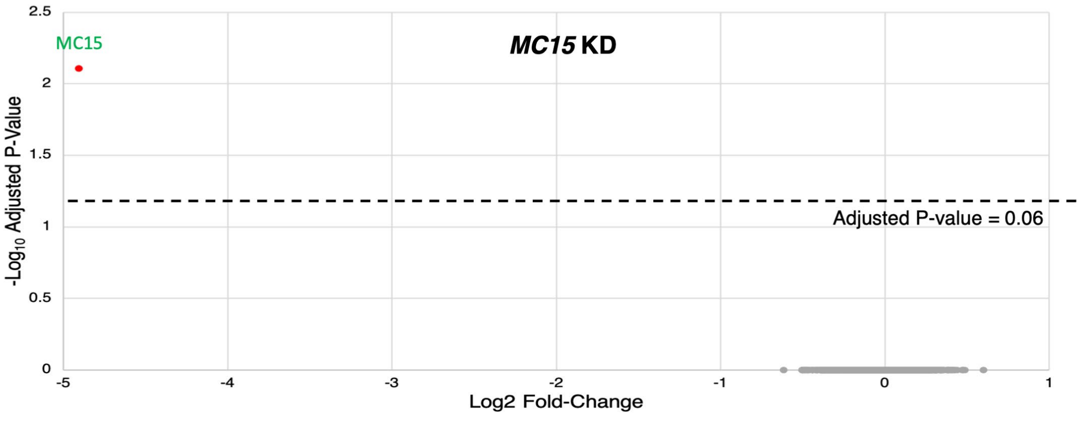

MC15 KD
MC15
Adjusted P-value = 0.06
-Log10 Adjusted P-Value
Log2 Fold-Change

Supplementary Figure 8. Knockdown of individual *MC*s affects only the targeted MC. Parasites were grown in the presence of tetracycline to induce knockdown of the indicated *MC*s. Demembranated flagella were purified from the indicated KD parasites or their respective NG-tagged parental cell lines (Ctrl) and subjected to TMT quantitative proteomic analysis. Volcano plots show significance (-Log_{10} Adjusted P-value) vs. relative abundance (Log_2 Fold-Change) for all proteins quantified by TMT proteomics. Adjusted P-value is from a moderated two-sided t-test, adjusted for multiple comparisons using Benjamini-Hochberg method. Relative abundance is calculated as *MC* KD/Ctrl. Results are from two independent biological samples. Proteins that met the filtering criteria (2-fold reduced, adjusted P-value $\leq$0.06; Supp. Dataset 1) are indicated by red dots. **a** *MC3* KD. **b** *MC5* KD. **c** *MC8* KD. **d** *MC15* KD.

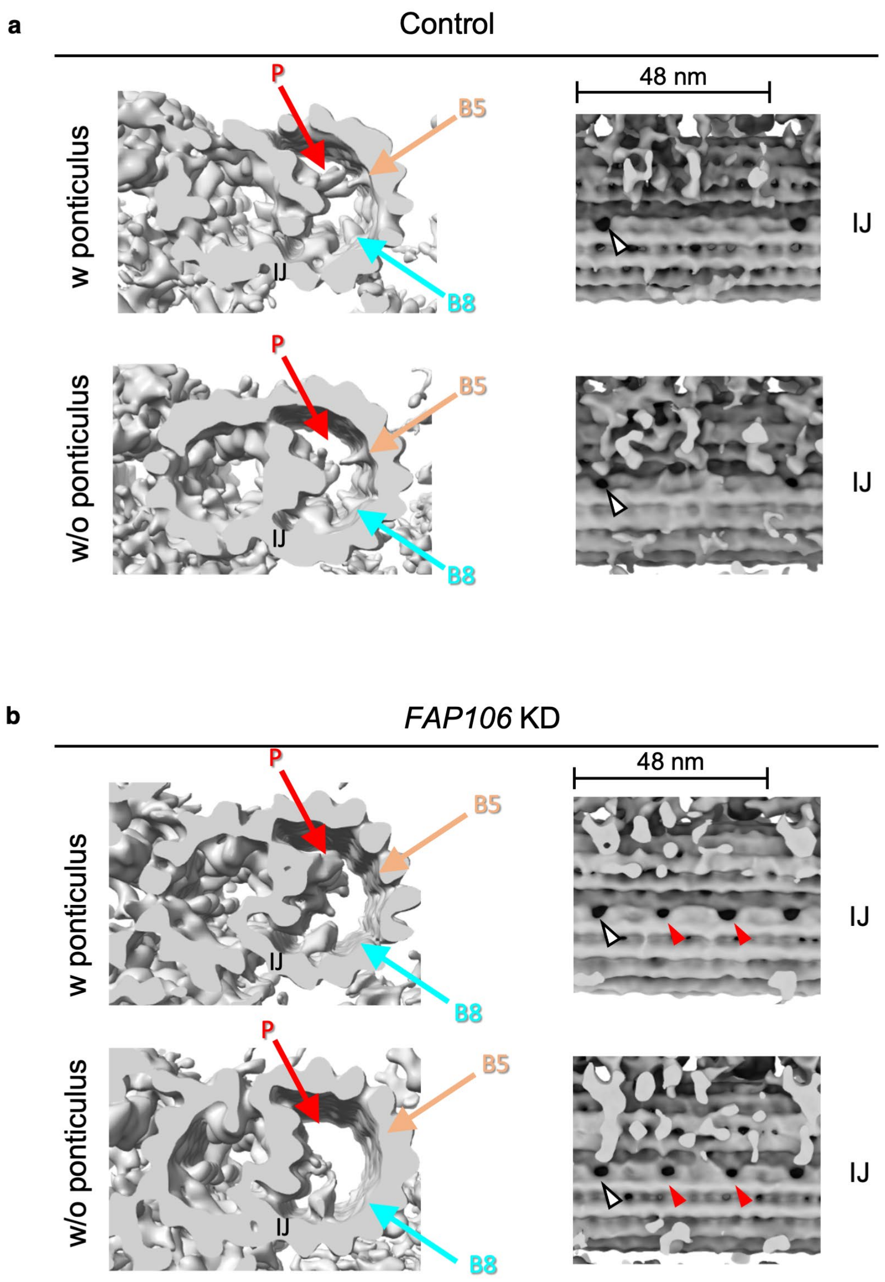

Supplementary Figure 9. Sub-tomogram averages from single flagella. Sub-tomogram averaging was performed using particles from individual flagella in **a** control or **b** *FAP106* KD samples. Each averaged tomogram shows the average obtained with particles from only a single flagellum. Individual flagella with (w) or without (w/o) clear ponticulus densities (red arrows) were identified as examples of old and new flagella, respectively, in both samples. White arrowheads show the inner junction (IJ) hole found in control samples. Loss of MIP B5 (peach arrow), MIP B8 (teal arrow), and appearance of extra IJ holes (red arrowheads) are dependent upon loss of FAP106, and do not show age-dependent differences. Number of particle images used for each averaged structure are listed in Supp. Table 1.

Supplementary Table 1. CryoET data collection statistics

	Control dataset 1	*FAP106* KD	*MC8* KD	Control dataset 2
**Data collection and processing**				
Microscope	Titan Krios	Titan Krios	Titan Krios	Titan Krios
Magnification	x53,000	x53,000	x53,000	x53,000
Voltage (kV)	300	300	300	300
Total Electron exposure (e–/Å2)	110	110	110	110
Slit width (eV)	20	20	20	20
Detector	K3	K3	K3	K3
Defocus range (µm)	-3.5 to -5.5	-3.5 to -5.5	-3.5 to -5.5	-3.5 to -5.5
Pixel size (Å)	1.69	1.69	1.69	1.69
Tilt-series increment	±2°	±2°	±2°	±2°
Tilt-series scheme	dose-symmetric*	dose-symmetric*	dose-symmetric*	dose-symmetric*
Tilt-series range	±60°	±60°	±60°	±60°
Tilt-series collected	52	51	69	17
Tilt-series used	20	18	52	9
**Data processing**				
Software tilt-series alignment	IMOD[#]	IMOD[#]	IMOD[#]	IMOD[#]
Software final reconstruction	PEET[&]	PEET[&]	PEET[&]	PEET[&]
Initial particle images (no.)	1616	1014	2626	626
Final particle images (no.)	1014	728	1936	538
# of particle images used for structure with ponticulus in Supp. Fig. 9	72	50	na	na
# of particle images used for structure without ponticulus in Supp. Fig. 9	64	60	na	na
Pixel size final reconstruction (Å)	6.76	6.76	6.76	6.76
Map resolution (Å)	18.5	19.5	16.5	24
FSC threshold	0.143	0.143	0.143	0.143

*Dose-symmetrical tilt-scheme as described in [7]

[#]IMOD [8]

[&]PEET [9]

Supplementary Table 2: Primers

		**Restriction Site**	**Primer Sequence**
FAP106 (KD)	Forward	XbaI	GCTCTAGACCAGCAAAGAAACCAACGGG
FAP106 (KD)	Reverse	HindIII	CCCAAGCTTACTGCATTCAGCTCCTTCCC
FAP106 (qRT PCR)	Forward	-	ACCTGATCGAGCCAGATGTG
FAP106 (qRT PCR	Reverse	-	ATTCTCGCCGCCTACGTTTG
RPS23 (qRT PCR) [10]	Forward	-	AGATTGGCGTTGGAGCGAAA
RPS23 (qRT PCR) [10]	Reverse	-	GACCGAAACCAGAGACCAGCA
MC3 (downstream sgRNA)	Forward	-	gaaattaatacgactcactataggGTCATCTGACTTACACTGATgttttagagctagaaatagc
G00 (sgRNA) [11]	Reverse	-	aaaagcaccgactcggtgccactttttcaagttgataacggactagccttattttaacttgctatttctagctctaaaac
MC3 (NG repair template)	Forward	-	CACTTTTGTCATCGAATCGTATATGACTACcttGTCTCGAAAGGTGAGGA
MC3 (NG repair template)	Reverse	-	ATACGTCAACCATATCCTTTTGTCTCAGTGccaatttgagagacctgtgc
MC3 (KD)	Forward	XbaI	GCTCTAGAAAAAACAGAACGTGACGCGG
MC3 (KD)	Reverse	HindIII	CCCAAGCTTTATTCGTTGGGCTGTGCTGT
MC5 (NG)	Forward	-	ACTTACACGCAGTGGCGTCTAACGATACCTACAGGCCGGGAGTCCACAACGTTAGCAAGTTTGGGGAAACATCGAGCAAGGGTTCTGGTAGTGGTTCCGG
MC5 (NG)	Reverse	-	CAATCCCCTCCTCTCTACTGTTTCTGTACATTTGCTTCCTCTGGTGATTGGCGATCATCTAGTGATGTTGTTATTTGAAGCCAATTTGAGAGACCTGTGC
MC5 (KD)	Forward	XbaI	GCTCTAGACACAAGCCAAAGTTCCACGG
MC5 (KD)	Reverse	HindIII	CCCAAGCTTTGCTAACGTTGTGGACTCCC
MC8 (NG)	Forward	-	TATATGTGGCGGGTCGGAAAGAAAACCGACGAGCCGCAACACTATCAAAGAATAGAGTTATAGCAACTTTTTATGGTGACCTTGTCTCGAAAGGTGAGGA

MC8 (NG)	Reverse	-	ACTTGCTGGAGGCAGCTTTATCCACACACAACCGCACAGACTTTCCTCTTGTGTTCTCTGTCCCAAATCTCACCCACTCACCAATTTGAGAGACCTGTGC
MC8 (KD)	Forward	Xbal	GCTCTAGAAACTTGCAACGCAGCATCAG
MC8 (KD)	Reverse	HindIII	CCCAAGCTTTAACCAAGTACTGCCAGCGG
MC15 (NG)	Forward	-	CAAAAGGTGCGACTGGTGTCTGGAAACCCAATACATTTGAATGCACAAGTGTAGTTCAATCATGTTTGCGCCGTTTTTATCTTGTCTCGAAAGGTGAGGA
MC15 (NG)	Reverse	-	AGCAAAAACTACGCACGCAGCCGCCCATTTGAAGCGCTACGCAACCTCCTCTTGCCTTATTCTAAGACATTAGGCTCTACCCAATTTGAGAGACCTGTGC
MC15 (KD)	Forward	Xbal	GCTCTAGAGGACACCATTCCGTCTCGTT
MC15 (KD)	Reverse	HindIII	CCCAAGCTTGAGGTTTAGGAGGCGCAAGT
FAP45 (APEX)	Forward	-	GAAGGAGTTAGAGGAACTCGGTGTGCCTGAAGAGTACTGCCAGGCACTGCAGAAGAAGATGAAGGTCAAGGTGGCCCGGCGAGGTTCTGGTAGTGGTTCC
FAP45 (APEX)	Reverse	-	AGGGAAAAAAGAGTGACTAGGGCAATACCAAAAATATAATGCGCGTATTAATACAAAAGTTCCTGCGGTTAAAAATGTAACCAATTTGAGAGACCTGTGC
FAP52 (APEX)	Forward	-	ACGGCAAGAAAATTGTGTCCGTTGGTGACGAAGGCGCTATTATGATTTGGTCTGTCTGTGACTTGGAGTTTAAGACGCTGGGTTCTGGTAGTGGTTCC
FAP52 (APEX)	Reverse	-	ACACACATACACACACACATACATATATTTATAATATAGAGCGTCAAAGGGGAGGAGTGCACGCACATAGATAAATAGGACCAATTTGAGAGACCTGTGC

Supplementary Methods

APEX2-dependent proximity labeling and purification of biotinylated proteins

APEX proximity labeling identifies proteins in proximity to an APEX-tagged protein, “the bait”, based on biotinylation and subsequent streptavidin purification and proteomic identification [6, 12]. We used as bait, two MIP proteins known to be inside the B-tubule of the DMT, FAP45 and FAP52, and one axonemal protein known to be outside the DMT, DRC1 (Supp. Fig. 6). APEX2-dependent biotinylation was performed as described [6] with the following modifications. For fluorescence microscopy, cells were biotinylated as described, then fixed with 0.2% paraformaldehyde. Cover slips were blocked in Dulbecco’s phosphate buffered saline (DPBS) + 8% normal donkey serum (NDS) + 2% BSA for 1 h at room temperature before incubation with 1:1000 anti-PFR primary antibody [13] overnight at 4°C. 1:200 streptavidin-Alexa 594 (Invitrogen S32356) was added during the incubation with 1:1500 donkey anti-rabbit Alexa 488 (Invitrogen A21206) secondary antibody. After post-fixation of coverslips with 4% PFA, coverslips were washed three times in DPBS. Images were acquired on a Zeiss Axioskop II microscope with Axiovision software and processed with Zen software. For shotgun proteomics, 2e8 cells were resuspended at 2e7 cells/mL in growth medium supplemented with 5 mM biotin-phenol for 1h. Cells were treated with 50 μM H_2O_2 for 1 min before quenching. After two washes with quenching solution, cells were washed with DPBS and resuspended at 2e8 cells/mL in Lysis buffer (PEME buffer + 1% NP40 + 4x Sigmafast EDTA-free protease inhibitor cocktail). 40 units of Turbo DNase (Thermo Fisher Scientific) were added and the samples were incubated at room temperature for 15 min. After centrifugation, the detergent-insoluble pellet was washed once in Lysis buffer and boiled for 5 min in Lysis buffer + 1% SDS. Purification of biotinylated proteins with streptavidin beads was performed as described [6]. Two independent biological replicates were prepared and analyzed by shotgun proteomics for each target protein (FAP45-APEX, FAP52-APEX, DRC1-APEX).

Shotgun proteomics

<u>*Sample Digestion and Desalting*</u>: The proteins bound to streptavidin beads were resuspended in digestion buffer (8M urea, 0.1 M Tris, pH 8.5) and reduced and alkylated via sequential 20-minute incubations of 5mM TCEP 10mM iodoacetamide at room temperature in the dark while being mixed at 1200 rpm in an Eppendorf thermomixer. Proteins were then digested by the addition of 0.1µg Lys-C (FUJIFILM Wako Pure Chemical Corporation, 125-05061) and 0.8µg Trypsin (Thermo Scientific, 90057) while shaking at 37°C overnight. The digestions were quenched via addition of formic acid to a final concentration of 5% (v/v). Each sample was desalted via C18 tips (Thermo Scientific, 87784) and then resuspended in 5% formic acid before analysis by LC-MS/MS.

<u>*LC-MS Acquisition and Analysis*</u>: Peptide samples were separated on a 75uM ID x 25cm C18 column packed with 1.9µm C18 particles (Dr. Maisch GmbH) using a 140-minute gradient of increasing acetonitrile and eluted directly into a Thermo Orbitrap Fusion Lumos mass spectrometer where MS/MS spectra were acquired by Data Dependent Acquisition (DDA). Database search was performed by using ProLuCID [14] and DTASelect2 [15, 16] implemented in Integrated Proteomics Pipeline IP2 (Integrated Proteomics Applications) and searched against a user assembled database consisting of all protein entries from the TriTrypDB [https://tritrypdb.org/tritrypdb/app] [17] for *T. brucei* strain 927 (version 7.0). A PSM-level false positive rate cutoff was set at 1% as estimated by a target-decoy database competition strategy, protein and peptide identifications were filtered by DTASelect2 and a minimum of two unique peptides per protein were required for the confident protein identification. Raw proteomic data have been deposited to the Mass Spectrometry Interactive Virtual Environment (MassIVE) under accession ID MSV000090660 [https://massive.ucsd.edu/ProteoSAFe/dataset.jsp?accession=MSV000090660].

Filtering criteria to identify MIP candidates (MCs)

For comparison of proteins identified in MIP (FAP45-APEX and FAP52-APEX), and non-MIP control (DRC1-APEX) samples, proteomics data were parsed using the Integrated Proteomics Pipeline IP2. The output from the Protein Identification STAT Compare tool (IDSTAT_COMPARE) in IP2 was filtered in Excel (Supp. Dataset 2). We first compared relative abundance for proteins in the MIP vs DRC1 bait samples. The threshold abundance ratio for inclusion as a MIP candidate is based on the average FAP45/DRC1 and FAP52/DRC1 ratios obtained for other known MIPs. Additional filters were subsequently applied to identify the most promising MIP candidates (MCs). In summary, proteins are identified as MCs based on: i) average FAP45/DRC1 and FAP52/DRC1 ratio meeting MIP threshold in APEX2 proteomics experiments, ii) similar abundance to known MIPs in APEX2 proteomics experiments, iii) localization to the flagellum from published data [18, 19], iv) absence of prior functional annotation, and v) lack of homologs outside kinetoplastids. The exact filtering criteria are as follows and in Supp. Dataset 2.

i. Average FAP45/DRC1 and FAP52/DRC1 ratio meeting MIP threshold in APEX2 proteomics experiments: Average of FAP45/DRC1 and FAP52/DRC1 abundance ratios (Avg MIP/DRC1 ratio) $\geq$ 1.235, where 1.24 is the lowest average abundance ratio observed for known B-tubule MIPs, considering PACRGs as B-tubule MIPs based on their exposure to the B-tubule lumen, but excluding FAP20, which for unknown reasons did not show enrichment in MIP samples relative to the non-MIP control.

ii. Relative abundance comparable to known B-tubule MIPs in APEX2 proteomics experiments: Average abundance in FAP45-APEX samples $\geq$ 0.00126 and average abundance in FAP52-APEX samples $\geq$ 9.59e-4, where 0.0013 is the lowest average abundance observed for known B-tubule MIPs (as defined in i.) in the FAP45-APEX samples and 9.59e-4 is the lowest average abundance observed for known B-tubule MIPs (as defined in i.) in the FAP52-APEX samples.

iii. Flagellum localization according to Tryptag [http://tryptag.org/] [18, 19]

iv. No homolog in *Chlamydomonas* or *Tetrahymena* based on OrthoMCL and/or reciprocal BLAST search
v. Not previously characterized (annotated as hypothetical or domain of unknown function)

Based on these criteria, we identified 15 MIP Candidates (MCs), named in no particular order (Supp. Dataset 2).

Fluorescence microscopy

Detergent-extracted cytoskeletons were prepared on cover slips as described for measurement of flagellum length. Cover slips containing cytoskeletons were rinsed in PEME and mounted onto slides for NeonGreen (NG) fluorescence microscopy. Immunofluorescence microscopy of biotinylation in whole cells was performed as described in [6]. Images were acquired on a Zeiss Axioskop II fluorescence microscope with a Plan-Apochromat 100x/1.4 objective lens and Axiovision software or on a Zeiss Axio Imager Z1 fluorescence microscope with a Plan-Apochromat 63x/1.4 objective lens and Zen software.

Supplementary References

1. Gui, M., et al., *De novo identification of mammalian ciliary motility proteins using cryo-EM.* Cell, 2021. **184**(23): p. 5791-5806.e19.
2. Khalifa, A.A.Z., et al., *The inner junction complex of the cilia is an interaction hub that involves tubulin post-translational modifications.* Elife, 2020. **9**.
3. Kubo, S., et al., *Native doublet microtubules from Tetrahymena thermophila reveal the importance of outer junction proteins.* Nat Commun, 2023. **14**(1): p. 2168.
4. Ma, M., et al., *Structure of the Decorated Ciliary Doublet Microtubule.* Cell, 2019. **179**(4): p. 909-922.e12.
5. Imhof, S., et al., *Cryo electron tomography with volta phase plate reveals novel structural foundations of the 96-nm axonemal repeat in the pathogen.* Elife, 2019. **8**.
6. Vélez-Ramírez, D.E., et al., *APEX2 Proximity Proteomics Resolves Flagellum Subdomains and Identifies Flagellum Tip-Specific Proteins in Trypanosoma brucei.* mSphere, 2021. **6**(1).
7. Hagen, W.J.H., W. Wan, and J.A.G. Briggs, *Implementation of a cryo-electron tomography tilt-scheme optimized for high resolution subtomogram averaging.* J Struct Biol, 2017. **197**(2): p. 191-198.
8. Kremer, J.R., D.N. Mastronarde, and J.R. McIntosh, *Computer visualization of three-dimensional image data using IMOD.* J Struct Biol, 1996. **116**(1): p. 71-6.
9. Heumann, J.M., A. Hoenger, and D.N. Mastronarde, *Clustering and variance maps for cryo-electron tomography using wedge-masked differences.* J Struct Biol, 2011. **175**(3): p. 288-99.
10. Lopez, M.A., E.A. Saada, and K.L. Hill, *Insect stage-specific adenylate cyclases regulate social motility in african trypanosomes.* Eukaryot Cell, 2015. **14**(1): p. 104-12.
11. Shaw, S., et al., *A transient CRISPR/Cas9 expression system for genome editing in Trypanosoma brucei.* BMC Res Notes, 2020. **13**(1): p. 268.
12. Hung, V., et al., *Spatially resolved proteomic mapping in living cells with the engineered peroxidase APEX2.* Nat Protoc, 2016. **11**(3): p. 456-75.
13. Saada, E.A., et al., *Insect stage-specific receptor adenylate cyclases are localized to distinct subdomains of the Trypanosoma brucei Flagellar membrane.* Eukaryot Cell, 2014. **13**(8): p. 1064-76.
14. Xu, T., et al., *ProLuCID: An improved SEQUEST-like algorithm with enhanced sensitivity and specificity.* J Proteomics, 2015. **129**: p. 16-24.
15. Cociorva, D., L.T. D, and J.R. Yates, *Validation of tandem mass spectrometry database search results using DTASelect.* Curr Protoc Bioinformatics, 2007. **Chapter 13**: p. Unit 13 4.
16. Tabb, D.L., W.H. McDonald, and J.R. Yates, 3rd, *DTASelect and Contrast: tools for assembling and comparing protein identifications from shotgun proteomics.* J Proteome Res, 2002. **1**(1): p. 21-6.
17. Aslett, M., et al., *TriTrypDB: a functional genomic resource for the Trypanosomatidae.* Nucleic Acids Res, 2010. **38**(Database issue): p. D457-62.
18. Billington, K., et al., *Genome-wide subcellular protein map for the flagellate parasite Trypanosoma brucei.* Nat Microbiol, 2023. **8**(3): p. 533-547.
19. Dean, S., J.D. Sunter, and R.J. Wheeler, *TrypTag.org: A Trypanosome Genome-wide Protein Localisation Resource.* Trends Parasitol, 2017. **33**(2): p. 80-82.
