# Supplementary material for: FAP106 is an interaction hub for assembling microtubule inner proteins at the cilium inner junction
Source: Nat Commun. 2023 Aug 26;14:5225. doi: 10.1038/s41467-023-40230-z (PMC10460401; doi:10.1038/s41467-023-40230-z)
Supplement: Supplementary file 3 — Description of Additional Supplementary Files [file 41467_2023_40230_MOESM3_ESM.docx]

**Description of Additional Supplementary Files**

**Supplementary Movie 1**

**Subtomographic average of the 48-nm repeat.** Video showing 3D reconstruction of 48-nm repeat from 29-13 (Control) and *FAP106* KD DMTs, rotating between views shown in Fig 2**b**-**e**.

**Supplementary Dataset 1**

**TMT proteomics data comparing detergent-extracted flagellum skeletons.** Each experiment is shown on a separate tab: *FAP106* KD vs 29-13 (Ctrl); *MC3* KD vs *MC3-NG*; *MC5* KD vs *MC5-NG*; *MC8 KD* vs *MC8-NG*; *MC15 KD* vs *MC15-NG*.

**Supplementary Dataset 2**

**APEX2-based proximity proteomics data and analysis.** ‘Unfiltered data’ tab contains the full list of proteins identified. Columns in ‘Filtered data’ tab shows filters used for assigning MIP candidates (MC1-15). ‘MC List’ tab lists MC1-15 gene IDs and Avg MIPs/DRC1 ratios.
