# Supplementary material for: FAP106 is an interaction hub for assembling microtubule inner proteins at the cilium inner junction
Source: Nat Commun. 2023 Aug 26;14:5225. doi: 10.1038/s41467-023-40230-z (PMC10460401; doi:10.1038/s41467-023-40230-z)
Supplement: Supplementary file 7 — Reporting Summary [file 41467_2023_40230_MOESM7_ESM.pdf]

nature portfolio

Corresponding author(s): Z. Hong Zhou, Kent L. Hill

Last updated by author(s): Jun 27, 2023

Reporting Summary

Nature Portfolio wishes to improve the reproducibility of the work that we publish. This form provides structure for consistency and transparency in reporting. For further information on Nature Portfolio policies, see our Editorial Policies and the Editorial Policy Checklist.

Statistics

For all statistical analyses, confirm that the following items are present in the figure legend, table legend, main text, or Methods section.

n/a	Confirmed	
☐	☒	The exact sample size (*n*) for each experimental group/condition, given as a discrete number and unit of measurement
☐	☒	A statement on whether measurements were taken from distinct samples or whether the same sample was measured repeatedly
☐	☒	The statistical test(s) used AND whether they are one- or two-sided<br>*Only common tests should be described solely by name; describe more complex techniques in the Methods section.*
☒	☐	A description of all covariates tested
☐	☒	A description of any assumptions or corrections, such as tests of normality and adjustment for multiple comparisons
☐	☒	A full description of the statistical parameters including central tendency (e.g. means) or other basic estimates (e.g. regression coefficient) AND variation (e.g. standard deviation) or associated estimates of uncertainty (e.g. confidence intervals)
☐	☒	For null hypothesis testing, the test statistic (e.g. *F*, *t*, *r*) with confidence intervals, effect sizes, degrees of freedom and *P* value noted<br>*Give P values as exact values whenever suitable.*
☒	☐	For Bayesian analysis, information on the choice of priors and Markov chain Monte Carlo settings
☒	☐	For hierarchical and complex designs, identification of the appropriate level for tests and full reporting of outcomes
☐	☒	Estimates of effect sizes (e.g. Cohen's *d*, Pearson's *r*), indicating how they were calculated

Our web collection on statistics for biologists contains articles on many of the points above.

Software and code

Policy information about availability of computer code

Data collection	Fluorescence microscopy images were collected with Zeiss software (Axiovision 4.8 or Zen 2.3). Dark field images for motility analyses were collected using Zeiss Zen 2.6 Pro. EM tilt series were collected using SerialEM 3.8.
Data analysis	FiJi 2.1.0/1.53c or 1.53t was used for flagellum length measurements, followed by statistical analysis in Graphpad Prism 7. Motility analysis was performed using a Matlab script described in Shimogawa et al. 2018 [doi: 10.1038/s41598-018-27228-0]. TMT proteomics data were analyzed using MaxQuant v. 1.6.17.0 and MSstatsTMT v. 1.6.6 Bioconductor package. APEX2 proteomics data were analyzed using Integrated Proteomics Pipeline IP2 and Microsoft Excel 2016. CryoET data was processed with MotionCor, Imod 4.9 and PEET 1.15.0 following the workflow described in Methods. CryoET data was visualized and analyzed with Imod 4.9 and ChimeraX 1.4. Protein modeling was performed using AlphaFold Colab [https://colab.research.google.com/github/deepmind/alphafold/blob/main/notebooks/AlphaFold.ipynb]

For manuscripts utilizing custom algorithms or software that are central to the research but not yet described in published literature, software must be made available to editors and reviewers. We strongly encourage code deposition in a community repository (e.g. GitHub). See the Nature Portfolio guidelines for submitting code & software for further information.

Data

Policy information about availability of data

All manuscripts must include a data availability statement. This statement should provide the following information, where applicable:

- Accession codes, unique identifiers, or web links for publicly available datasets
- A description of any restrictions on data availability
- For clinical datasets or third party data, please ensure that the statement adheres to our policy

The structure data generated during the current study have been deposited in the Electron Microscopy Data Bank (EMDB) repository, with the accession codes EMD-28802 [https://www.ebi.ac.uk/emdb/EMD-28802], EMD-28803 [https://www.ebi.ac.uk/emdb/EMD-28803], EMD-28804 [https://www.ebi.ac.uk/emdb/EMD-28804], and EMD-28805 [https://www.ebi.ac.uk/emdb/EMD-28805]. The raw proteomics data generated during the current study have been deposited to the Mass Spectrometry Interactive Virtual Environment (MassIVE) repository under accession IDs MSV000090660 [https://massive.ucsd.edu/ProteoSAFe/dataset.jsp?accession=MSV000090660] and MSV000090661 [https://massive.ucsd.edu/ProteoSAFe/dataset.jsp?accession=MSV000090661] and the analysis tables are provided as Supplementary Datasets. The structure of the DMT from Bos taurus shown in Supp. Fig. 3 is available in the EMDB repository, accession code EMD-24664 [https://www.ebi.ac.uk/emdb/EMD-24664] and the atomic model is available as PDB:7rro [https://doi.org/10.2210/pdb7RRO/pdb]. All other data supporting the findings of this study are available within the paper or its supplementary information files. T. brucei genome and protein sequences are publicly available at TriTrypDB [https://tritrypdb.org/tritrypdb/app] and protein localizations are publicly available on TrypTag [http://tryptag.org/].

Research involving human participants, their data, or biological material

Policy information about studies with human participants or human data. See also policy information about sex, gender (identity/presentation), and sexual orientation and race, ethnicity and racism.

Reporting on sex and gender	N/A
Reporting on race, ethnicity, or other socially relevant groupings	N/A
Population characteristics	N/A
Recruitment	N/A
Ethics oversight	N/A

Note that full information on the approval of the study protocol must also be provided in the manuscript.

Field-specific reporting

Please select the one below that is the best fit for your research. If you are not sure, read the appropriate sections before making your selection.

[x] Life sciences [ ] Behavioural & social sciences [ ] Ecological, evolutionary & environmental sciences

For a reference copy of the document with all sections, see nature.com/documents/nr-reporting-summary-flat.pdf

Life sciences study design

All studies must disclose on these points even when the disclosure is negative.

Sample size	No sample size calculation was performed. Sample sizes were chosen based on previous studies (Shimogawa et al., 2018; Imhof et al., 2019), best practices in the field, and practicality.
Data exclusions	No data were excluded.
Replication	Growth curves were performed in triplicate. qRT-PCR was performed in duplicate on three independent RNA samples. Motility analyses were performed on two independent biological samples. Proteomics analyses were performed on two independent biological replicates. Micrographs are representative of results from at least two independent experiments. For cryoET, where replicates were not feasible, the data are representative of the average. All attempts at replication were successful, with variability shown.
Randomization	Randomization was not relevant for the experiments performed here. Experiments involved comparing cell lines (mutant vs control) under identical treatment conditions and sample preparation procedures, processed in parallel.
Blinding	Blinding was not relevant for this study. Test groups (mutant or control cell lines) were processed through identical analysis pipelines in parallel. Although researchers were not blinded to cell line identity during experiments, the major conclusions of this study are based on data that were acquired and/or analyzed using automated protocols and software.

Reporting for specific materials, systems and methods

We require information from authors about some types of materials, experimental systems and methods used in many studies. Here, indicate whether each material, system or method listed is relevant to your study. If you are not sure if a list item applies to your research, read the appropriate section before selecting a response.

Materials & experimental systems

n/a	Involved in the study
☐	☒ Antibodies
☐	☒ Eukaryotic cell lines
☒	☐ Palaeontology and archaeology
☒	☐ Animals and other organisms
☒	☐ Clinical data
☒	☐ Dual use research of concern
☒	☐ Plants

Methods

n/a	Involved in the study
☒	☐ ChIP-seq
☒	☐ Flow cytometry
☒	☐ MRI-based neuroimaging

Antibodies

Antibodies used	rabbit anti-PFR2 (custom antibody generated by Kent Hill lab; Saada et al., 2014 [https://doi.org/10.1128/ec.00019-14]), donkey anti-rabbit Alexa 594 (Invitrogen A21207); streptavidin-Alexa 594 (Invitrogen S32356), donkey anti-rabbit Alexa 488 (Invitrogen A21206)
Validation	anti-PFR2 antibody has been validated on T. brucei cells by IFA and by Western blotting on purified protein and cell lysates. Commercially available antibodies have been validated by the supplier.

Eukaryotic cell lines

Policy information about cell lines and Sex and Gender in Research

Cell line source(s)	29-13 T. brucei cell line obtained originally from George Cross (Wirtz et al., 1999) [https://doi.org/10.1016/S0166-6851(99)00002-X]
Authentication	Genes are periodically sequenced for various projects.
Mycoplasma contamination	Not tested for mycoplasma
Commonly misidentified lines (See ICLAC register)	None
